# Effect of Case Discussion with Expert Centers on Treatment Outcome of Intrahepatic Cholangiocarcinoma

**DOI:** 10.1245/s10434-026-19540-1

**Published:** 2026-04-12

**Authors:** Thomas C. Zwaan, Stefan Buettner, Lydia G. van der Geest, Joanne Verheij, Andries E. Braat, Joris I. Erdmann, Jeroen Hagendoorn, Judith de Vos-Geelen, Bas Groot Koerkamp, Pim B. Olthof

**Affiliations:** 1 Department of Surgery, Erasmus MC Cancer Institute, Rotterdam, The Netherlands; 2 Department of Research and Development, Netherlands Comprehensive Cancer Organisation (IKNL), Utrecht, The Netherlands; 3 Department of Pathology, Amsterdam UMC, Amsterdam, The Netherlands; 4 Department of Surgery, Leiden University Medical Center, Leiden, The Netherlands; 5 Department of Surgery, Amsterdam UMC, Amsterdam, The Netherlands; 6 Department of Surgery, UMC Utrecht, Utrecht, The Netherlands; 7 Department of Internal Medicine, Division of Medical oncology, GROW–Research Institute for Oncology & Reproduction, Maastricht UMC+, Maastricht, The Netherlands; 8 Department of Hepatobiliary, Transplant and Endocrine Surgery, Universiteitsziekenhuis Antwerpen, Antwerp, Belgium

**Keywords:** Intrahepatic cholangiocarcinoma, Expert centers, Multidisciplinary team discussion, Biliary tract cancer, Case discussion

## Abstract

**Background:**

Intrahepatic cholangiocarcinoma (iCCA) is a rare liver malignancy with poor prognosis. This study assessed how case discussion of patients at expert centers within multidisciplinary team meetings (MDTs) is associated with treatment and survival in iCCA patients in the Netherlands.

**Methods:**

This retrospective cohort study analyzed data registered in the Netherlands Cancer Registry between 2017 and 2021 on all consecutive patients diagnosed with iCCA. Data included patient demographics, tumor characteristics, and the involvement of a member of an expert center at case discussions. Outcomes included overall survival and treatment type, which was specified as tumor-directed (e.g., resection, ablation, systemic treatment, or other treatment modalities) vs. supportive care only.

**Results:**

Among the 1.622 patients included, median overall survival was 5.4 months, and 825 (50.9%) underwent tumor-directed therapy. Patients discussed at expert centers (59.6%) were more likely to receive tumor-directed therapy (58.8% vs. 37.2%, *P* < 0.001) and undergo surgery (20.3% vs. 5.7%, *P* < 0.001). In the multivariable analysis, patients discussed at expert centers had better overall survival (hazard ratio 0.69 (0.62–0.78)).

**Conclusions:**

Centralized care through MDT discussion at expert centers was associated with increased likelihood of receiving tumor-directed therapy and improved survival in iCCA patients. This study highlights the potential for improved patient outcomes through structured expert consultations.

**Supplementary Information:**

The online version contains supplementary material available at 10.1245/s10434-026-19540-1.

In the Western world, intrahepatic cholangiocarcinoma (iCCA) is the second most common primary malignancy of the liver after hepatocellular carcinoma, with an incidence of one to two patients per 100,000 person-years.^1^ Although the incidence of iCCA is rising, it remains a rare disease with approximately 400 new patients annually in the Netherlands.^2^ Usually, iCCA is asymptomatic or nonspecific in presentation and therefore often diagnosed at an advanced stage, frequently presenting as large, multifocal, or metastatic disease, limiting treatment options. Untreated, survival is poor with a median overall survival of 5 months.^3^ In the absence of locally advanced or metastatic disease, surgical resection offers a chance of long-term survival, but only about 15% of all patients qualify for resection.^4^

The Netherlands has a universal healthcare system, in which liver surgery is conducted in 22 hospitals, each performing a minimum of 20 procedures annually. Given the low incidence of iCCA, a centralized approach of care for iCCA patients in the Netherlands is already a reality, and oncological guidelines recommend treatment of these patients in a limited number of expert centers. Despite widespread regional collaboration in cancer networks and centralization, a recent study showed a 33% resection rate when patients were first diagnosed with iCCA in academic centers versus only 13% in nonacademic centers.^4^ This nationwide study assesses regional collaboration in iCCA care by expert multidisciplinary team (MDT) discussions, which may influence treatment decisions and outcomes.

## Methods

All patients diagnosed with iCCA between 2017 and 2021 and registered in the Netherlands Cancer Registry (NCR) were included. Patients were notified to the NCR by means of the Dutch national pathology archives (PALGA) and the Dutch National Hospital Care Registration (LBZ; hospital discharges and outpatient visits). All included patients were verified in patient records in Dutch hospitals by trained registration clerks approximately 9 to 12 months after diagnosis. Tumor location and type were coded according to the International Classification of Diseases for Oncology (ICD-O-3) with invasive C22.1 intrahepatic bile duct (adeno)carcinoma selected for this study. Follow-up and survival data were obtained through annual linkage of the NCR with Dutch civil municipal registry. The study protocol was evaluated by the Dutch Hepatocellular & Cholangiocarcinoma Group (DHCG, 2023-06) and the Privacy Board of the NCR (K23.174). Medical ethical approval and informed consent was not required in accordance with Dutch law.

Patient characteristics retrieved from the NCR consisted of age, sex, and tumor-specific variables. Tumor staging was based on the cTNM stage (UICC-TNM, 8th edition, 2017 onwards). The hospital of diagnosis was classified as either an expert or nonexpert center, based on the first hospital where the patient presented with symptoms of the tumor regardless of where treatment was ultimately received. Expert centers for primary liver tumors in the Netherlands are the eight academic hospitals. In the Netherlands, eight oncology networks were identified (blinded as regions A till H), each region consisted of 0–2 expert centers and 4–16 referring hospitals. An expert MDT discussion was defined as a (regional) MDT meeting that included in-person or virtual participation of a physician from an expert center. Participating specialties included HPB-surgeons, medical oncologists, hepatologists, radiotherapists, and interventional radiologists. Tumor-directed therapy consisted of surgery, (palliative) systemic therapy, radiotherapy, ablation, or local chemotherapy. A major resection was defined as resection of ≥3 Couinaud liver segments.^4^ The most recent update of the survival data was on February 1, 2023. Survival time was defined as the interval between diagnosis and death or last follow-up on February 1, 2023.

### Statistical Analysis

Categorical variables were presented as numbers with percentages and the differences between these variables were evaluated using the chi-square or Fisher’s exact test. Continuous variables were presented as median value with interquartile range (IQR) and differences between variables were tested by using Mann-Whitney *U* tests. Univariable and multivariable analysis were conducted using Cox proportional hazard regression. Variables with a *p*-value below 0.2 at univariable analysis were included in the multivariable analysis. Hazard ratios and survival data were presented with their 95% confidence interval (CI). All statistical analyses were performed with IBM SPSS for Windows version 28 (released 2021, Armonk, NY).

## Results

Between 2017 and 2021, a total of 1,622 patients were diagnosed with iCCA in the Netherlands. Median age was 70 years (interquartile range [IQR] 61–78) and 824 patients (50.8%) were female. At time of diagnosis, 762 patients (47%) had metastatic disease (i.e., stage IV). Tumor-directed therapy was given in 825 patients (50.9%). The most common reasons for withholding therapy were poor patient condition (*n* = 285, 17.6%), patient preference (n = 244, 15%), and rapid disease progression or limited life expectancy (n = 195, 12%). Patient, tumor, and treatment characteristics are shown in Table 1.

**Table 1:** Hospital of initial presentation. Patient, disease, and treatment characteristics of all patients according to the hospital type (expert vs. nonexpert centers)

	Total cohort N = 1622	Nonexpert N = 1385	Expert N = 237	*P*
Age*, median (IQR)*	70 (61-78)	70 (62-76)	68 (60-74)	0.003
Female sex	824 (50.8)	701 (50.6)	123 (51.9)	0.726
*CT stage*				
cT1	325 (20.0)	262 (18.9)	63 (26.6)	<0.001
cT2	830 (51.2)	729 (52.6)	101 (42.6)	
cT3	70 (4.3)	62 (4.5)	8 (3.4)	
cT4	194 (12.0)	164 (11.8)	30 (12.7)	
cTx	203 (12.5)	168 (12.1)	35 (14.8)	
*cN stage*				
cN0	798 (49.2)	669 (48.3)	129 (54.4)	0.021
cN1	692 (42.7)	605 (43.7)	87 (36.7)	
cNx	132 (8.1)	111 (8.0)	21 (8.9)	
Multifocal disease in the liver	728 (44.9)	653 (47.1)	75 (31.6)	<0.001
cM1 stage	762 (47.0)	683 (49.3)	79 (33.5)	<0.001
Discussed at any MDT meeting	1224 (75.5)	1031 (74.4)	193 (81.4)	0.022
Discussed with physician of expert center present (in-person or online)	966 (79.9)	785 (56.7)	181 (76.3)	<0.001
Biliary drainage	204 (12.6)	188 (13.6)	16 (6.8)	0.003
*Hospital of treatment*				
Nonexpert center	1041 (64.2)	1034 (74.7)	7 (3.0)	<0.001
Expert center	581 (35.8)	351 (25.3)	230 (97.0)	
Tumor-directed therapy	825 (50.9)	670 (48.4)	155 (65.4)	<0.001
*Reason no tumor directed therapy*				
Patient condition	285 (17.6)	256 (18.5)	29 (12.2)	<0.001
Patient wishes	244 (15.0)	219 (15.8)	25 (10.5)	
Rapid progression or short life expectancy	195 (12.0)	181 (13.1)	14 (5.9)	
No symptoms	3 (0.2)	3 (0.2)	–	
Other or unknown reason	70 (4.3)	56 (4.0)	14 (5.9)	
*Treatment*				
Surgery	247 (15.2)	167 (12.1)	80 (33.8)	<0.001
Palliative chemotherapy	503 (31.0)	450 (32.5)	53 (22.4)	0.013
Radiotherapy	29 (1.8)	21 (1.5)	8 (3.4)	0.046
Ablation	14 (0.9)	7 (0.5)	7 (3.0)	<0.001
Local chemotherapy	32 (2.0)	24 (1.7)	8 (3.4)	0.123

*IQR* interquartile range; *MDT* multidisciplinary team

Of all patients, 1,385 (85.4%) were first diagnosed in nonexpert centers, and 237 (14.6%) patients directly presented in expert centers. Patients diagnosed in expert centers were younger (median age 68 years [IQR 60–74] vs. 70 years [IQR 62–76], *P* = 0.003) and had a lower prevalence of metastatic disease (33.5% vs. 49.3%, *P* < 0.001). Following diagnosis in an expert center, more patients underwent tumor-directed therapy (65.4% vs. 48.4%, *P* < 0.001), and the resection rate was higher (33.8% vs. 12.1%, *P* < 0.001).

Among patients diagnosed in a nonexpert center, 785 (56.7%) were discussed in the presence of an expert from an expert center during the MDT (i.e., a physician from an expert center participates in a formal MDT, either in-person or online), compared with 181 (76.4%) of the patients diagnosed in an expert center (*P* < 0.001). Characteristics of patients based on whether they were discussed with an expert center are shown in Table 2. Patients who were not discussed in an expert MDT were more likely to have metastatic disease (58.1% vs. 39.9%, *P* < 0.001), less likely to receive tumor directed therapy (37.2% vs. 58.8%, *P* < 0.001), and had lower rates of surgery (5.7% vs. 20.3%, *P* < 0.001).

**Table 2:** Patient discussed with expert center. Patient, disease, and treatment characteristics based on whether cases were discussed with an expert center

	Discussed with expert center			
	Yes	No	Unknown	*P*
	N = 966	N = 576	N= 80	
Age*, median (IQR)*	68 (60–74)	68 (60–74)	70 (61–76)	<0.001
Female sex	514 (53.2)	277 (48.1)	33 (41.3)	0.058
*cT stage *				
*cT1*	225 (23.3)	82 (14.2)	19(23.8)	<0.001
*cT2*	491 (50.8)	303 (52.6)	36 (45.0)	
*cT3*	35 (3.6)	32 (5.6)	3 (3.8)	
*cT4*	125 (12.9)	58 (10.1)	11 (13.8)	
*cTx*	90 (9.3)	101 (17.5)	11 (13.8)	
*cN stage *				
cN0	513 (53.1)	248(43.1)	37 (43.8)	<0.001
cN1	402 (41.6)	255 (44.3)	35 (43.8)	
cNx	51 (5.3)	73 (12.7)	8 (10.0)	
Multiple lesions	396 (41.0)	299 (51.9)	33 (41.3)	<0.001
cM1 stage	385 (39.9)	335 (58.1)	42 (52.5)	<0.001
Discussed at MDT meeting	966 (100.0)	178 (30.9)	80 (100.0)	<0.001
Biliary drainage	141 (14.6)	54 (9.4)	9 (11.3)	0.003
*Hospital of diagnosis*				
Nonexpert center	785 (81.3)	531(92.2)	69 (86.3)	<0.001
Expert center	181 (18.7)	45 (7.8)	11 (13.8)	
*Hospital of treatment*				
Nonexpert center	457 (48.3)	511 (88.7)	63 (78.8)	<0.001
Expert center	499 (51.7)	65 (11.3)	17 (21.3)	
Tumor-directed therapy	568 (58.8)	214 (37.2)	43 (53.8)	<0.001
*Reason no tumor directed therapy*				
Patient condition	137 (14.9)	135 (23.4)	13 (16.3)	
Patient wishes	136 (14.8)	94 (16.3)	14 (17.5)	
Rapid progression or short life expectancy	84 (9.1)	104 (18.1)	7 (8.8)	
No symptoms	2 (0.2)	1 (0.1)	–	
Other or unknown reason	39 (4.0)	28 (4.9)	3 (3.8)	
*Treatment*				
Surgery	196 (20.3)	33 (5.7)	18 (22.5)	<0.001
Palliative chemotherapy	313 (32.4)	166 (38.8)	24 (25.3)	0.003
Radiotherapy/radioembolization	26 (2.7)	2 (0.3)	1 (1.3)	<0.001
Ablation	12 (1.2)	2 (0.3)	-	0.073
Local chemotherapy	31 (3.2)	-	1 (1.3)	<0.001

*IQR* interquartile range; *MDT* multidisciplinary team

**Fig. 1:**
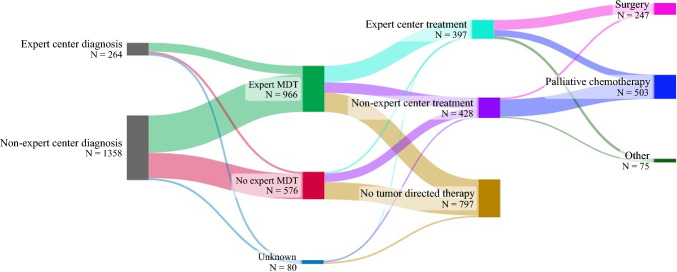
Sankey plot illustrating type of treatment in patients with iCCA without metastasis based on the hospital of diagnosis, expert MDT discussion, and type of hospital of treatment

The treatment of patients without metastasis based on the hospital of diagnosis, expert MDT discussion, and hospital of treatment is shown in Fig. 1. Of the 860 patients without metastasis, 241 (28%) were not discussed in an expert MDT. Patient condition, patients’ refusal, or rapid progression or short life expectancy was reported as reason for not initiating treatment in 148 of these patients (61.4%). Among the remaining 93 patients, 15 were aged 80 or older, and 27 had multiple lesions.

**Fig. 2:**
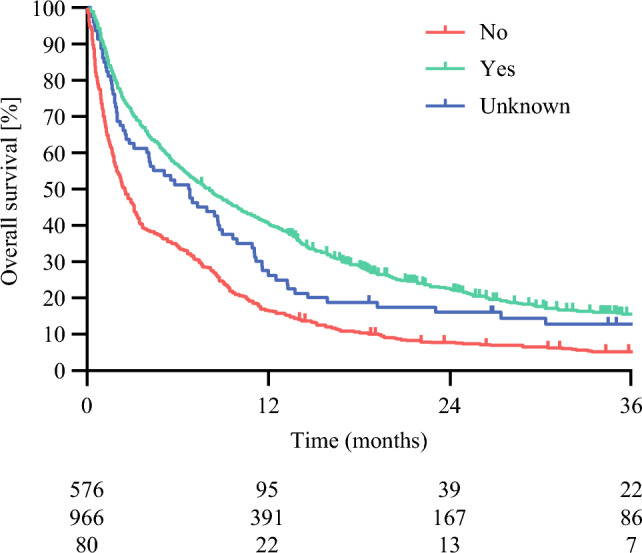
Kaplan-Meier curves for overall survival based on patients discussed with an expert center

At the end of follow-up, 1,417 patients (77.4%) had died. Among survivors, the median follow-up was 39.7 months (95% confidence interval [CI] 36.1–42.3). Median overall survival was 5.4 months (95% CI 4.7–6.1). Median overall survival was 8.1 (95% CI 7.0–9.2) months in those discussed in an expert MDT versus 2.4 (95% CI 1.9–2.9) months in those who were not (*P* < 0.001; Fig. 2). In multivariable analysis, after adjustment for age, female sex, clinical node status, multiple lesions, metastatic disease, and biliary drainage, expert MDT discussion was associated with improved overall survival (HR 0.69; 95% CI 0.62–0.78; Table 3).

**Table 3:** Uni- and multivariable Cox regression analysis for overall survival

	Univariable analysis		Multivariable analysis	
	Hazard ratio (95%CI)	*P*	Hazard ratio (95%CI)	*P*
*Discussed with expert center*				
No	Reference		Reference	
Yes	0.53 (0.47–0.59)	<0.001	0.69 (0.62–0.78)	<0.001
Unknown	0.64 (0.50–0.81)	0.031	0.77 (0.60–0.99)	0.044
Age	1.02 (1.01–1.02)	<0.001	1.02 (1.02–1.03)	<0.001
Female sex	0.86 (0.78–0.96)	0.006	0.91 (0.82–1.01)	0.073
*Clinical node status*				
cN0	Reference		Reference	
cN+	1.82 (1.63–2.04)	<0.001	1.47 (1.31–1.66)	<0.001
Unknown	1.90 (1.57–2.30)	<0.001	1.44 (1.18–1.76)	<0.001
*Multiple lesions*				
Single	Reference		Reference	
Multiple	2.17 (1.91–2.46)	<0.001	1.73 (1.51–1.98)	<0.001
Missing	1.61 (1.39–1.86)	<0.001	1.37 (1.18–1.60)	<0.001
Metastatic disease	2.06 (1.85–2.30)	<0.001	1.60 (1.42–1.80)	<0.001
Biliary drainage	1.54 (1.33–1.80)	<0.001	1.56 (1.34–1.82)	<0.001

*CI* confidence interval

### Surgery

Of the 247 patients who underwent surgical resection, 209 underwent surgery in an expert center (84.6%). The probability of a negative margin was 72.4% in expert centers versus 60% in nonexpert centers (*P* = 0.421). Ninety-day mortality was 6.2% in expert centers and 10.5% in nonexpert centers (*P* = 0.307) Two of 16 patients (12.5%) died following minor liver resection in a nonexpert center, whereas there was no postoperative mortality after 67 minor liver resections in expert centers (*P* = 0.035). Regarding negative margins in major liver resections, a difference could not be demonstrated when comparing nonexpert centers to expert centers (71.3% vs. 47.4%, *P* = 0.091). During minor liver surgery, negative margin rates were 75% in both expert centers and nonexpert centers (*P* = 0.960). Ninety-day mortality after major liver resection was 9.0% in expert centers and 10.5% in non-expert centers (*P* = 0.688). Median overall survival was 41.5 (29.2–53.8) months in expert versus 32.4 (10.8–54.0) months in nonexpert centers (*P* = 0.77).

### Regional Differences

**Fig. 3:**
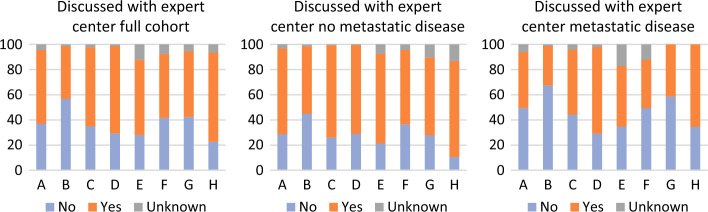
Regional differences in case discussion

Across the eight regional cancer care collaboratives in the Netherlands, the proportion of patients not discussed with an expert center ranged from 22.7% to 56.8% (*P* < 0.001). For patients without metastatic disease, this ranged from 10.5% to 44.9% (*P* < 0.001), and from 29.1% to 67.5% for patients with metastatic disease (*P* < 0.001) (Fig. 3).

The proportion of patients who underwent tumor-directed therapy did not vary across the regions of diagnosis (*P* = 0.119). Resection rate in patients without metastatic disease ranged from 21.7% to 38% across regions (*P* = 0.107). The proportion of patients with stage I-III iCCA without a resection but who received palliative systemic therapy ranged from 14.7% to 30.4% across regions (*P* = 0.056). In patients with stage IV iCCA, the proportion of patients with palliative systemic therapy ranged from 28.2 to 52.2% (*P* = 0.023) (supplemental Fig. 1).

Characteristics of surgical treatment differed across regions (Table 4). The majority of patients underwent surgery in an expert center (*n* = 209, 84.6%). Differences in 90-day mortality across regions could not be demonstrated (0–13.2%, *P* = 0.653). However, the proportion of negative margins did differ across regions (36.4–94.4%, *P* < 0.001).

**Table 4:** Regional differences in surgical treatment and outcomes

		Region of treatment								
		A	B	C	D	E	F	G	H	*P*-value
Patients	1622	225	144	280	300	199	175	134	165	
Resections	247	38	13	55	62	24	20	13	22	
*Location*										
Expert	84.6%	94.7%	61.5%	63.6%	95.2%	79.2%	100%	92.3%	90.9%	<0.001
Non-expert	15.4%	5.3%	38.5%	36.4%	4.8%	20.8%	0%	7.7%	9.1%	
*Type of resection*										
Major	64.7%3	71.1%	75.0%	58.8%	59.7%	63.6%	77.5%	23.1%	81.8%	<0.001
Minor	35.3%	23.7%	25%	41.2%	40.3%	36.4%	12.5%	76.9%	18.2%	
*Negative margins*										
Overall	70.5%	59.5%	36.4%	75.5%	86.3%	45.5%	94.4%	69.2%	63.4%	<0.001
Expert	72.4%	60%	37.5%	84.8%	87.5%	38.9%	94.4%	75%	60.0%	<0.001
Nonexpert	60%	50%	33.3%	60%	66.7%	75%	–	0%	100%	0.95
*90-day mortality*										
Overall	6.9%	13.2%	0.0%	7.3%	6.5%	4.2%	10.0%	0%	4.5%	0.653
Major	9.2%	18.5%	0.0%	6.7%	0.8%	7.1%	6.7%	0%	5.6%	0.692

## Discussion

This nationwide study of 1,622 patients diagnosed with iCCA in the Netherlands in 2017–2021 demonstrated an association between case discussion with an expert center and overall survival (HR 0.72 [0.64–0.81], *P* < 0.001). Furthermore, a higher resection rate in patients first diagnosed in expert centers (33.8% vs. 12.1%) was seen, and patients who were discussed in an expert MDT were more likely to receive tumor-directed therapy (58.8% vs. 37.2%) or a resection (20.3% vs. 5.7%).

Our findings underscore the importance of case discussion in an expert MDT, which was independently associated with improved overall survival in this study. To our knowledge, this study is the first report on an association between case discussion in an expert MDT and outcomes for patients with iCCA. A review on the value of MDT in cancer care showed that, although the evidence is variable, overall implementation of MDTs lead to better outcomes for cancer patients. Potential explanations include better diagnostics (especially in patients with iCCA, which is often misclassified as adenocarcinoma of unknown primary), staging, treatment selection and delivery, and higher rates of participation in clinical trials.^5–8^ While the association in our study between expert MDT discussion and improved survival is statistically significant, a closer examination of the Kaplan-Meier curves reveals that the greatest survival benefit occurs within the first few months following diagnosis. This early difference raises the possibility that patients discussed in an expert MDT may have been selected based on factors, such as better performance status or more favorable tumor characteristics, which could influence early survival outcomes. Variations in management between centers might arise from differences in judgement regarding resectability, drainage options and, local (e.g., SIRT) and systemic treatment, which might differ between surgeons performing these operations themselves and those who do not. The importance of involvement of experts in treating iCCA is also reflected in both the National Comprehensive Cancer Network (NCCN) guideline on hepatobiliary cancers (version 2.2021) and in the European Society for Medical Oncology (ESMO) guideline on biliary tract cancers. The NCCN guideline recommends multidisciplinary evaluation of patients to optimize treatment.^9^ The ESMO guideline recommends to discuss all patients eligible for resection, with recurrent disease, or liver-confined iCCA in a MDT.^10^

Indeed, in this study, a tendency toward more tumor-directed therapies was observed in cases discussed in an expert MDT. This finding extends previous evidence by suggesting that discussion within an expert MDT may represent an important factor in the process of making the right diagnosis, staging iCCA, and initiating the appropriate treatment. Earlier studies have shown that patients diagnosed in expert centers are more likely to receive curative-intent treatment (i.e., resection) compared with those diagnosed in non-expert centers.^11^ A previous study in the Netherlands showed a resection rate of 33% for patients diagnosed with iCCA in expert centers versus 13% in nonexpert centers.^4^ The higher resection rate for patients diagnosed in expert centers was also observed for perihilar CCA.^12^ Likewise, Sutton et al. found that referral center treatment (i.e., expert care) was independently associated with tumor-directed therapy in iCCA: curative intent surgery was more frequent (OR 4.51 [2.89–7.01], *P* < 0.001) in expert centers compared with community center treatment.^13^ We found that, after diagnosis, referral for discussion in an expert MDT was associated with a higher chance of tumor-directed treatment. Most resections were performed in expert centers; however, in our study in seven of eight regions were resections also performed in nonexpert centers. This suggests that referral of all patients diagnosed in nonexpert centers to expert MDTs could improve treatment and outcome. In a recent retrospective cohort, patients with biliary tract cancer were more likely to receive adjuvant chemotherapy when they underwent resection in an expert center (48.7% vs. 43.4%; *P* < 0.001).^14^ In the multivariable analysis of this study, treatment at an academic medical center was an independent variable for receiving adjuvant chemotherapy, which is consistent with the broader observation in our study that expert care increases the likelihood of receiving tumor-directed therapy.^14^ A French nationwide study showed that chemotherapy and resection were performed most frequently in cancer centers and university hospitals respectively, which is consistent with our findings.^15^ Together with available literature, our study highlights the importance of involving physicians from expert centers in (regional) MDTs in the treatment of iCCA.

In this retrospective study, it is difficult to delineate the reasons why not all patients with localized disease were discussed in an expert MDT, as recommended by the Dutch national guideline. Some patients were reported to have rapid progression and/or short life expectancy and therefore were not expected to benefit from expert MDT discussion. Similarly, the majority of patients who did not undergo treatment owing to poor condition were unlikely to benefit from MDT discussion. However, it may be argued that some of these patients could potentially undergo some form of treatment with proper management and prehabilitation in an experienced center. Patients who chose not to undergo treatment were also likely to be in poor condition. Nevertheless, there might be some benefit in counseling by experienced CCA physicians. For the remaining patients who were not discussed in an expert MDT, the reasons are less clear. Some patients were older than 80 years, and others presented with multiple lesions, which may have influenced the decision not to refer. However, even these patients might have had multiple treatment options. For other patients, the underlying reasons for not referring cannot be determined based on the available data. A potential contributing factor may be unfamiliarity with the disease and available treatment options among referring physicians. Despite centralization of care in the Netherlands for iCCA palliative (systemic) treatment is usually available in nonexpert centers, which is likely the reasons why the majority of patients remained under care in nonexpert centers. Surgical care is only possible in 15% of patients with iCCA. These patients are usually treated in expert centers; yet this is only a minority of the cohort.

Numerous papers have identified a clear volume-outcome relationship in liver surgery.^16–18^ This is even more pronounced in complex liver surgery, such as for intrahepatic cholangiocarcinoma.^19^^,^^20^ In our study, surgery in an expert center was not associated with better 90-day mortality or higher R0 resection rate. Only in major liver resections, a higher R0 resection rate was observed after surgery in expert centers compared with nonexpert centers. Supporting these findings, Wu et al. (2019) reported higher rates of R0 resection in expert centers compared with community programs (72.4% vs. 67.7%, *P* = 0.006). Also, a longer median overall survival was observed when comparing expert centers to community programs (11 vs. 6 months, *P* < 0.001).^21^ Similarly, Lee et al. observed that expert centers had fewer positive surgical margins (21.4% vs. 26.1%, adjusted *P* = 0.04) and a reduced 90-day mortality rate (odds ratio [OR] 0.62, adjusted *P* = 0.01). ^22^

Regional variations in treatment and outcomes of iCCA in our study were observed. The location of surgery (expert vs. nonexpert center) varied across the regions in our study. Resections in our 4-year study cohort were performed in at least 15 centers. Within this study, the R0-resection rate varied between 33.3% and 100%, and 90-day mortality between 0 and 13.2%; however, these differences might arise owing to the small numbers per region and the amount of regions.

Such data may suggest that the centralization and volume of care facilities are crucial factors that can influence treatment efficacy and patient survival, emphasizing the potential for enhanced outcomes through concentrated specialist care. At the same time, strengthening regional cooperation between expert and nonexpert centers may further enhance survival, for instance by establishing regional care pathways, ensuring referral to expert MDTs to discuss the optimal treatment options and where to perform them, as well as foster collaborations for clinical trials.

This study has several limitations, most of which are due to the retrospective nature of this study. The NCR consists of limited in-depth data on surgical and systemic treatment, i.e., data on the reasons of unresectability are scarce. Furthermore, differences in resection rates between expert centers and nonexpert centers and between the regions could be partially due to differences in background liver disease, the stage of the disease, and the initial physical fitness of patients (i.e., performance status and comorbidities). In multivariable analyses, we could not adjust for these important factors, leading to residual confounding. Additionally, patients not being discussed in an expert MDT largely correlate with poor clinical condition (i.e., unfit for surgery), metastatic disease, or a nontreatment wish by the patient. In a real-world clinical setting, poor clinical condition and metastatic disease is often a reason for a nonexpert MDT to be cautious with extensive treatment options and might lead during the shared decision-making process to a decision to not refer the patient to expert centers. These decisions lead to selection bias and might partially explain our findings.

In this nationwide study of 1,622 patients diagnosed with iCCA in the Netherlands, a higher resection rate was observed in patients diagnosed in an expert center, tumor-directed therapy was started more often, and discussion with an expert center was an independent positive prognostic factor for overall survival. Furthermore, differences between regions regarding case management and treatment outcome for iCCA patients were observed, even in a small, urbanized country with centralized healthcare, such as the Netherlands. Based on these findings, we conclude that through strengthening centralization of care and (regional) collaboration between expert centers and nonexpert centers by improving the referral pathways and increase MDT participation, an improvement can be made in the quality of care for patients with iCCA.

## Supplementary Information

Below is the link to the electronic supplementary material.

Supplementary file1 (DOCX 69 KB)
