# Supplementary material for: Effect of Case Discussion with Expert Centers on Treatment Outcome of Intrahepatic Cholangiocarcinoma
Source: Ann Surg Oncol. 2026 Apr 12;33(7):6090–8. doi: 10.1245/s10434-026-19540-1 (PMC13242394; doi:10.1245/s10434-026-19540-1)

**Supplement(s) to Effect of Case Discussion with Expert Centers on Treatment Outcome of Intrahepatic Cholangiocarcinoma**

Supplemental Figure 1: Regional differences in tumor therapy


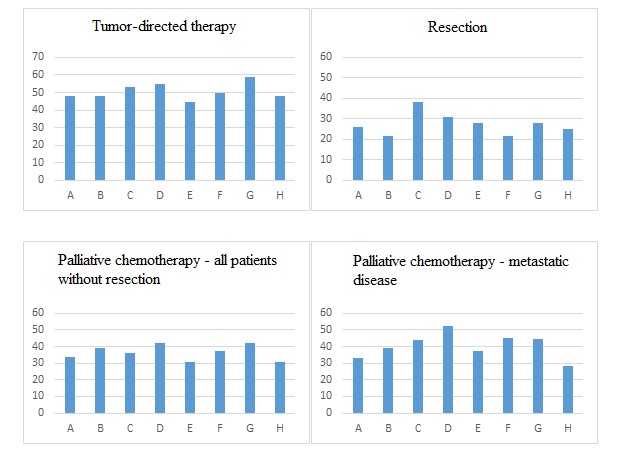

Supplement: Supplementary file 1 — Supplementary file1 (DOCX 69 KB) [file 10434_2026_19540_MOESM1_ESM.docx]
